# Metastatic Gastric Adenocarcinoma in the Inguinal Hernia Sac Diagnosed Radiologically: A Case Report

**DOI:** 10.34172/aim.28951

**Published:** 2024-04-27

**Authors:** Ahmed Said Çil, İbrahim Üntan

**Affiliations:** ^1^Department of Radiodiagnostic, Ahi Evran University, Faculty of Medicine, Kırşehir, Türkiye; ^2^Department of Urology, Ahi Evran University, Faculty of Medicine, Kırşehir, Türkiye

**Keywords:** Gastric adenocarcinoma, Inguinal hernia, Malignant ascites, Metastasis, Magnetic resonance imaging, Ultrasonography

## Abstract

Macroscopic tumor implants in the hernia sac are a very rare condition. They occur as a result of the implantation of malignant cells in the malignant ascites from the inguinal canal to the hernia sac. In this case report, we share the clinical and radiological findings of the macroscopic tumoral implants in the hernia sac at the level of the inguinal canal and scrotum in a male patient aged 65 years with a history of total gastrectomy for gastric adenocarcinoma and developing malignant ascites six months after the surgery.

## Introduction

 Intra-abdominal fluid accumulation accompan ying malignant tumors is called malignant ascites; in this situation, viable malignant cells in the fluid can be implanted all over the peritoneum. In patients with indirect inguinal hernia, malignant ascites may fill the hernia sac that extends to the scrotum. Since the hernia sac originates from the peritoneum, malignant cells that pass into the fluid from the primary tumor may also be implanted in the hernia sac wall. The diagnosis is usually made by pathological examination of the excised hernia sac. Here, we report the radiological findings of inguinal and intrascrotal macroscopic tumoral implants in the hernia sac of a patient who underwent total gastrectomy for gastric adenocarcinoma and developed malignant ascites and scrotal swelling 6 months after the operation.

## Case Report

**Figure 1:**
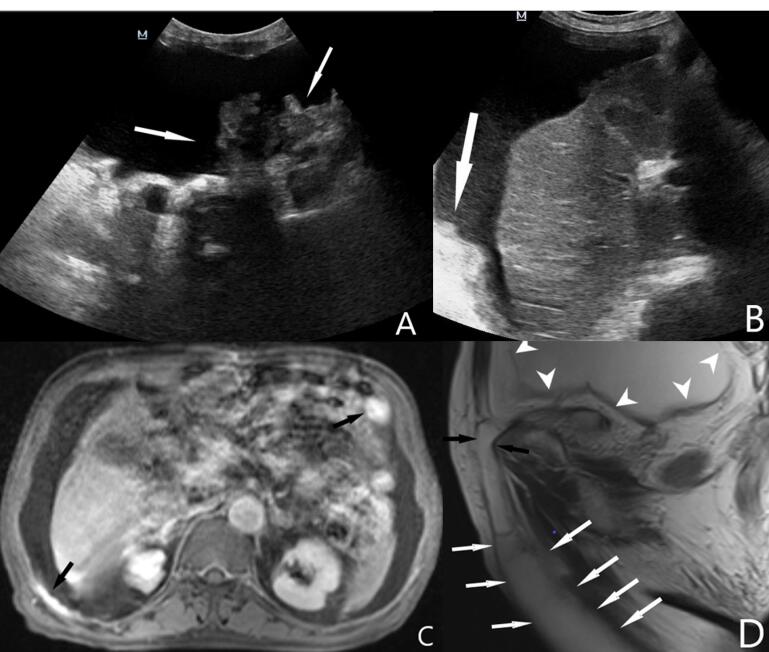
Ultrasonographic Images; Intraperitoneal fluid accumulation and peritoneal tumoral implants in the left **(A)** and right upper **(B)** quadrants. Contrast-enhanced axial magnetic resonance image **(C)** shows intraabdominal fluid and enhanced peritoneal implants (Black arrows). T2 weighted sagittal magnetic resonance image **(D)** shows intra-abdominal fluid (arrowheads), the hernia sac and fluid extension to the scrotum along the right inguinal canal (arrows)

**Figure 2:**
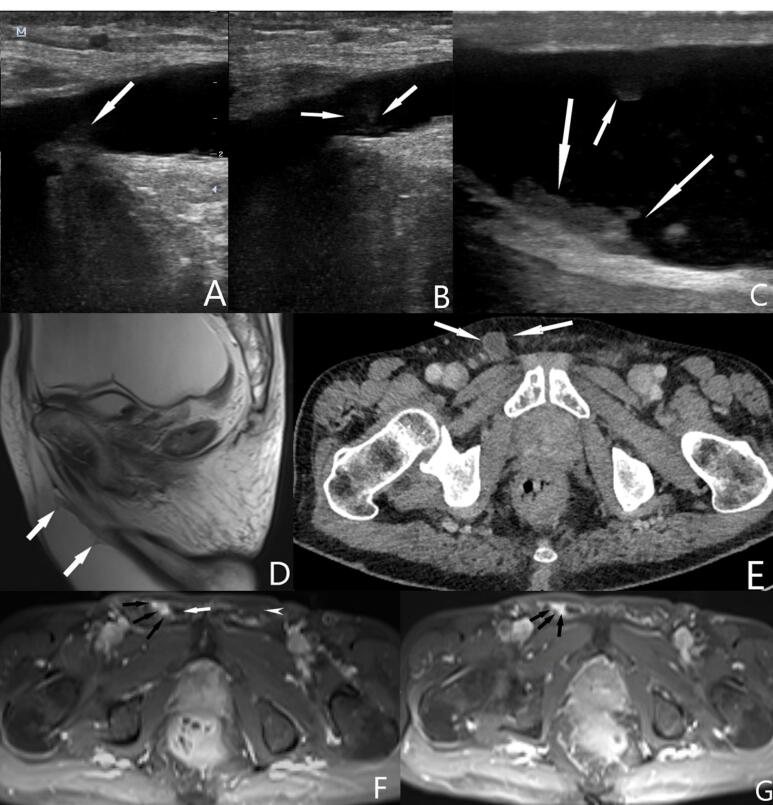
Ultrasonographic images show tumoral implants in the hernia sac at the level of the inguinal canal **(A, B)** and scrotum **(C).** T2 weighted sagittal magnetic resonance image **(D) **shows tumoral implants in the hernia sac (arrows). Contrast-enhanced axial CT image **(E) **shows hypodense fluid in the hernia sac in the right inguinal canal (white arrows). Contrast-enhanced axial magnetic resonance images **(F, G)** show enhanced tumoral implants in the hernia sac at the level of the inguinal canal (Black arrows). Note that contrast enhancement is not seen in the left inguinal canal (white arrowhead) and hypointense fluid (white arrow) is visible in the hernia sac **(F)**

 A 65-year-old male patient was admitted to the urology outpatient clinic because of right scrotal swelling. The patient had a history of surgery for gastric adenocarcinoma six months ago and ongoing chemotherapy. On ultrasound examination, there was extensive collection of fluid in the abdomen. The fluid extended to the scrotum along the right inguinal canal. Peritoneal implants due to dissemination of gastric adenocarcinoma were observed in different regions of the abdomen (Figure 1). Also, solid structures attached to the wall of the hernia were noticed at the level of the right inguinal canal and scrotum. Contrast-enhanced abdominal magnetic resonance imaging revealed contrast enhancement in the implants both in the abdomen and the hernia wall (Figure 2). Histopathological examination of the fluid aspirated from the inguinal canal revealed a malignant epithelial proliferation consisting of solid sheets of round to oval tumor cells with vesicular nuclei, prominent nucleoli, and some cells with signet ring-like aspects. The patient passed away within one month.

## Discussion

 Malignant ascites constitutes approximately one-tenth of all ascites cases; it commonly stems from endometrial, esophageal, colorectal, pancreatic, hepatobiliary, ovarian, breast, gastric, lung, and primary peritoneal carcinomas.^1^ Occasionally, internal malignancies manifest solely with ascites. The development of malignant ascites is closely associated with a poor prognosis, with the average survival reported at only four months.^2^ Several mechanisms are related to development of malignant ascites such as obstruction of draining lymphatics as a consequence of malignancy, direct production and secretion of fluid into the peritoneal cavity by highly active malignancies, and functional cirrhosis developing in patients with extensive hepatic metastases leading to portal hypertension.^3^ The cellular components of malignant ascites include tumoral cells, which may exist as individual cells or spheroids, and stromal cells, which comprise fibroblasts, endothelial cells, and inflammatory cells. Investigations into the role of ascitic fluid flow during intraperitoneal malignant seeding have revealed that ascitic fluid flow takes place along intra-abdominal specific pathways based on the contribution of factors such as subdiaphragmatic pressure and gravity.^4^ The development of peritoneal metastasis is a process that occurs as a result of the attachment of free tumor cells which were previously detached from the primary tumor and shed to peritoneal mesothelial cells, invasion of the attached tumor cells to basement membrane; and tumor growth with the onset of angiogenesis.^5^ In metastatic gastric cancer patients, direct seeding into the peritoneal cavity is reported to be more than 60%.^6^

 While peritoneal dissemination is expected to remain within the peritoneal cavity limited to the diaphragm, anterior abdominal wall, pelvic floor, and vertebrae, in patients with ascites, unexpected metastases may also occur in cases of herniation that confuses the cavity boundaries. Cancers have been reported in the literature that spread to the hernial sacs in the presence or absence of ascites, albeit limited. Tumors in the hernial sac occur in fewer than 0.5% of all surgically excised sacs. Hernial sac tumors are categorized into three types, and this classification is based on the anatomical proximity of the hernial sac and the tumor. Metastatic and primary tumors of the viscera incarcerated in the hernia sac constitute the intrasaccular type, metastatic and primary tumors of pleura and peritoneum residing in the hernia sac constitute the saccular type, and any tumors that protrude through the hernia defect out of the hernia sac constitute the extrasaccular type. The intrasaccular form is the most frequent type and the metastases that are distributed to the inguinal herniation area via ascitic fluid containing malignant cells belong to this type, as presented in the current case.

 The presence of tumors in hernia sacs is a very rare condition.^7^ Few cases of tumors in the inguinal hernia sac secondary to dissemination through the ascites due to primary malignancies have been reported in the literature. Although rare, all of the presentations were either examined during hernia surgery, diagnosed histopathologically after hernia surgery, or diagnosed in scintigraphic studies. However, our case uniquely represents hernial tumoral implantation diagnosed by radiological methods.^8^

 In a multiple case report, Lowenfels et al demonstrated metastasis of ovarian, colon, and prostate cancer to the inguinal hernia sac.^9^ Nicholson reported 15 cases of tumors detected during inguinal hernia repair, three of which were appendiceal, three ovarian, three peritoneal, two prostatic, two pancreatic, one rectal, and two of unknown origin.^10^ Matthews and McClelland reported a 75-year-old woman with a single ovarian cyst which was eventually diagnosed as a solitary necrotic omental metastasis from an ovarian cystadenocarcinoma that had herniated through the femoral canal.^11^ Brenner et al presented two men, aged 63 and 61, who were diagnosed with malignant mesothelioma based on nodules found during hernia repair and subsequent histopathological examination.^12^ Korn et al had a two-case presentation similar to our case. His initial case was a 68-year-old man who underwent surgery for right inguinal hernia; notable nodules were observed on the inner side of the hernia sac which were also noticed on the mesentery during hernia repair, and histopathological examination was reported as adenocarcinoma, but the patient deceased within six months before the primary focus was detected. The other case was a 62-year-old male who was operated on due to an irreducible inguinal mass seven months after subtotal gastrectomy for gastric cancer. He had metastatic implants in the mesenteric adipose tissue and a mini-laparotomy revealed extensive peritoneal tumor dissemination before the patient passed away within two months.^13^ Oruç reported a case of metastatic gastric cancer in the inguinal hernial sac confirmed by histopathological examination during inguinal hernia repair before the primary gastric tumor was detected.^14^ Takeuchi et al presented a malign mix mullerian tumor of the ovary growing into the inguinal hernial sac in a 77-year-old woman which was diagnosed using magnetic resonance imaging and revealed that ovarian tumors spread to inguinal hernial sacs more often.^15^ Díaz-Montes et al presented a 49-year-old postmenopausal woman with ascites and history of total abdominal hysterectomy who was diagnosed with an ovarian cancer recurrence in the inguinal hernia sac using computed tomography and ^18^ F-fluorodeoxyglucose positron emission tomography.^16^ Qin et al presented a 63-year-old woman who was incidentally diagnosed with bilateral femoral hernial metastases of adenocarcinoma from an undetermined primary focus.^17^ Nakayama et al presented a colon cancer recurrence disseminated into the peritoneal cavity including the right inguinal hernia sac of a 68-year-old man with ascites.^18^ Wang reported a case of a 70-year-old woman presenting with an incarcerated right inguinal hernia which was reported as a perivascular epitheloid cell tumor after the excision.^19^ Yokoyama et al presented a 78-year-old man with an irreducible inguinal hernia which contained a metastasis originating from cholangiocarcinoma discovered during herniorrhaphy.^8^ Brimo Alsaman et al presented a 39-year-old previously healthy man with gastric adenocarcinoma metastasis in the right inguinal hernia before deceasing within one week; in this case, the suspicion of inguinal herniation led the authors to perform a surgical intervention in which metastasis was found.^20^ This case was similar to ours in terms of gastric origin, presence of ascites and hernial sac metastasis, but in our case the metastatic implant was diagnosed utilizing magnetic resonance imaging and ultrasonography without a surgical intervention. Han et al reported a lung cancer metastasis in inguinal hernia sac which was detected utilizing ^18^ F fluorodeoxyglucose positron emission tomography/computed tomography before hernia repair.^21^ Gill-Wiehl and Veenstra reported a case of a man aged 83 years who was incidentally discovered to have a diagnosis of metastatic prostate cancer on pathology following elective inguinal hernia repair.^22^

 Tumoral implants in hernia sacs are sometimes in the form of visible macroscopic nodules, and sometimes in the form of occult implants at the microscopic level. For this reason, although some authors have recommended microscopic examination of all hernia sacs, the general belief is that microscopic examination should be performed only in selected risky cases.^10^ Most of the metastatic carcinoma cases in hernia sacs reported in the literature are cases diagnosed incidentally during hernial excision.^17^ As far as we know, this is the first case with radiological images of macroscopic implants secondary to gastric adenocarcinoma in the hernia sac.^14^

 Urologists generally recognize that not all scrotal swelling originates from the urogenital system and must keep in mind different and unexpected clinical aspects. Although it is considered not possible to see microscopic implants with radiological diagnostic methods, our case reveals that ultrasonography and magnetic resonance imaging are very sensitive in the diagnosis of macroscopic implants. The clinician should collaborate with the radiologist and consider ultrasonography and magnetic resonance imaging as alternatives to surgical sampling or nuclear medicine scans.
